# North American Douglas-fir (*P*. *menziesii)* in Europe: establishment and reproduction within new geographic space without consequences for its genetic diversity

**DOI:** 10.1007/s10530-019-02045-2

**Published:** 2019-07-08

**Authors:** Marcela van Loo, Desanka Lazic, Debojyoti Chakraborty, Hubert Hasenauer, Silvio Schüler

**Affiliations:** 1 Department of Botany and Biodiversity Research, Faculty of Life Sciences, University of Vienna, Rennweg 14, 1030 Vienna, Austria; 2 Department of Forest Growth and Silviculture, Austrian Research Centre for Forests BFW, Seckendorff-Gudent-Weg 8, 1131 Vienna, Austria; 3 Institute of Silviculture, University of Natural Resources and Life Sciences, Peter-Jordan Str. 82, 1190 Vienna, Austria

**Keywords:** Douglas-fir, Climate similarity, Population genetics, Natural regeneration, Inter-varietal hybridisation, Fine-scale spatial genetic structure

## Abstract

Genetic admixture and plasticity along with propagule pressure, large seed dispersal distances and fast adaptation support successful establishment and spread of introduced species outside their native range. Consequently, introductions may display climatic niche shifts in the introduced range. Douglas-fir, a controversial forest and ornamental conifer represented by two ecologically different and hybridising varieties, was transferred multiple times outside the native range in North America. Here, we compare climatic and genetic patterns of 38 native populations from North America with six old *Pseudotsuga menziesii* populations with natural regeneration in the introduced range in Central Europe. Following variety and geographic origin assessment of introduced populations, genotypic and climatic data were examined for signatures of inter-varietal gene flow, reduced genetic diversity, presence of fine-scale spatial genetic structure (SGS), dispersal patterns, and climate similarities between native and introduced range. In the introduced range, dominating coastal variety originated from a restricted area in the US, whereas the interior variety, with limited presence in the European sites, displayed wider geographic origin. Variety hybrids with contributing coastal, but not the interior parent were identified. Differences in genetic diversity between both ranges, but also among the parent and their respective offspring populations in Europe were not found. Old populations in general lacked any SGS, whereas natural regeneration revealed different patterns of SGS. Distances of propagule dispersal ranged between 2.5 and 92 m. The climate of the studied European introduced range was most similar to the climate of the coastal variety from the western Cascade range from which the majority of the analysed coastal European Douglas-fir, but not the European interior variety, was assigned to originate. The results we present here shed not only light on dynamics of invasive species in the introduced range in general, but also allow for refinement of climatic niche modeling when using lower than species level.

**Electronic supplementary material:**

The online version of this article (10.1007/s10530-019-02045-2) contains supplementary material, which is available to authorized users.

## Introduction

It is more often a rule rather than an exception that non-native species were introduced into new ranges multiple times and/or from different geographic and genetically differentiated source populations allowing for interactions not possible in the native range (e.g. Dlugosch and Parker 2008; Henry et al. 2009; Uller and Leimu 2011; Novo et al. 2015; Rijal et al. 2015). As indicated by a number of studies, intraspecific genetic admixture, i.e. blending and interbreeding of native source populations in the new distribution range, contributes significantly to the invasive success of non-native species (reviews of Prentis et al. 2008; Rius and Darling 2014). There is growing empirical evidence that admixed genotypes resulting from the intraspecific genetic admixture may also benefit from heterosis (phenotypic superiority of offspring genotypes compared to their parents) and contribute to biological invasions (van Kleunen et al. 2015; Hahn and Rieseberg 2017). On the long-term the genetic admixture boosts the genetic variation brought by introduction into the non-native range, providing a larger pool of raw material for adaptive evolution (Kolbe et al. 2004; Pairon et al. 2010; Rius and Darling 2014). Nevertheless, intra-varietal admixture and partitioning/changes of genetic variations are not explicitly associated with introduced ranges. We found them also in native areas when estimating genetic structure, which allow us to study events such as species demographic history, colonization out of refugia, past and present hybridization events (van Loo et al. 2015, van Boheemen et al. 2017).

Invasive species, similarly to native species, have to evolve in response to environment, in order to survive and succeed in both the native and introduced ranges. To understand the evolutionary mechanisms related to invasiveness such as the role of selection, admixture, and local adaptation of the introduced species, it is essential to know the source populations (areas) (Prentis et al. 2008; van Boheemen et al. 2017, 2019, Bouteiller et al. 2019). For ecological but also economic reasons, source populations and their geographic origin offer information on the adaptive and growth potential, on plasticity, tolerance to environmental stress, insects, and diseases (Morgenstern 1996). This also applies to widespread forest trees. Some of them differentiated in subspecies, varieties, or ecotypes as a result from population history and complex interactions with the environment allowing them to occupy a wide range of environmentally heterogeneous habitats (Hunt 1993).

Along with human activities, climate is a major factor affecting the distribution of species worldwide (Watt et al. 2010). The association between species distribution and climate has been explored intensively to predict current and future distributions of species within ecological niche models. However, introduced tree species are often known to diverge in their niche requirements when introduced to a new habitat (Randin et al. 2006; Camenen et al. 2016; Atwater et al. 2018). They may even display different and wider climatic tolerances in the new range than within their native distribution area (Molofsky et al. 2017; Atwater et al. 2018) despite the fact that initially introduced populations are often subjected to founder effects resulting in reduced genetic diversity (Rijal et al. 2015; Bouteiller et al. 2019).

In short-living species, the history of the introduction and fate of the introduced populations including the detection of geographic source populations, the interaction with the new environment and further species spread is difficult to reconstruct due to short life cycles and long introduction history. This is different for long-living organisms such as tree species, where many initially introduced and planted populations still exist, often with several generations of progeny in one place. In addition, several conifers e.g. *Pseudotsuga menziesii, Thuja plicata, Pinus ponderosa*, *Pinus contorta*, and *Abies grandis* are distributed far beyond their native ranges in both the Northern and Southern Hemisphere.

Across Europe there is hardly a tree species so controversial and caught between nature conservation and forestry as the North American Douglas-fir (*P. menziesii* (Mirb.), Franco). It is the most frequent introduced conifer in European forests (Köble and Seufert 2001) when we take into account number of European countries where it grows in forests (> than 30 countries), and the area it occupies in Europe. While it is viewed to be potentially invasive in Germany, Austria, Bulgaria, and Great Britain—Douglas-fir is considered to be invasive in New Zealand, Argentina and Chile in areas adjacent to plantations (Richardson and Rejmánek 2011). In addition, the species properties/characteristics portrayed in the previous paragraphs refer to this tree: it was introduced multiple times and from different geographic areas of its wide native distribution range, and it is represented by two ecologically distinct varieties (coastal and interior variety) which hybridise in the native range and produce inter-varietal admixed genotypes (Gugger et al. 2010; van Loo et al. 2015).

**Fig. 1:**
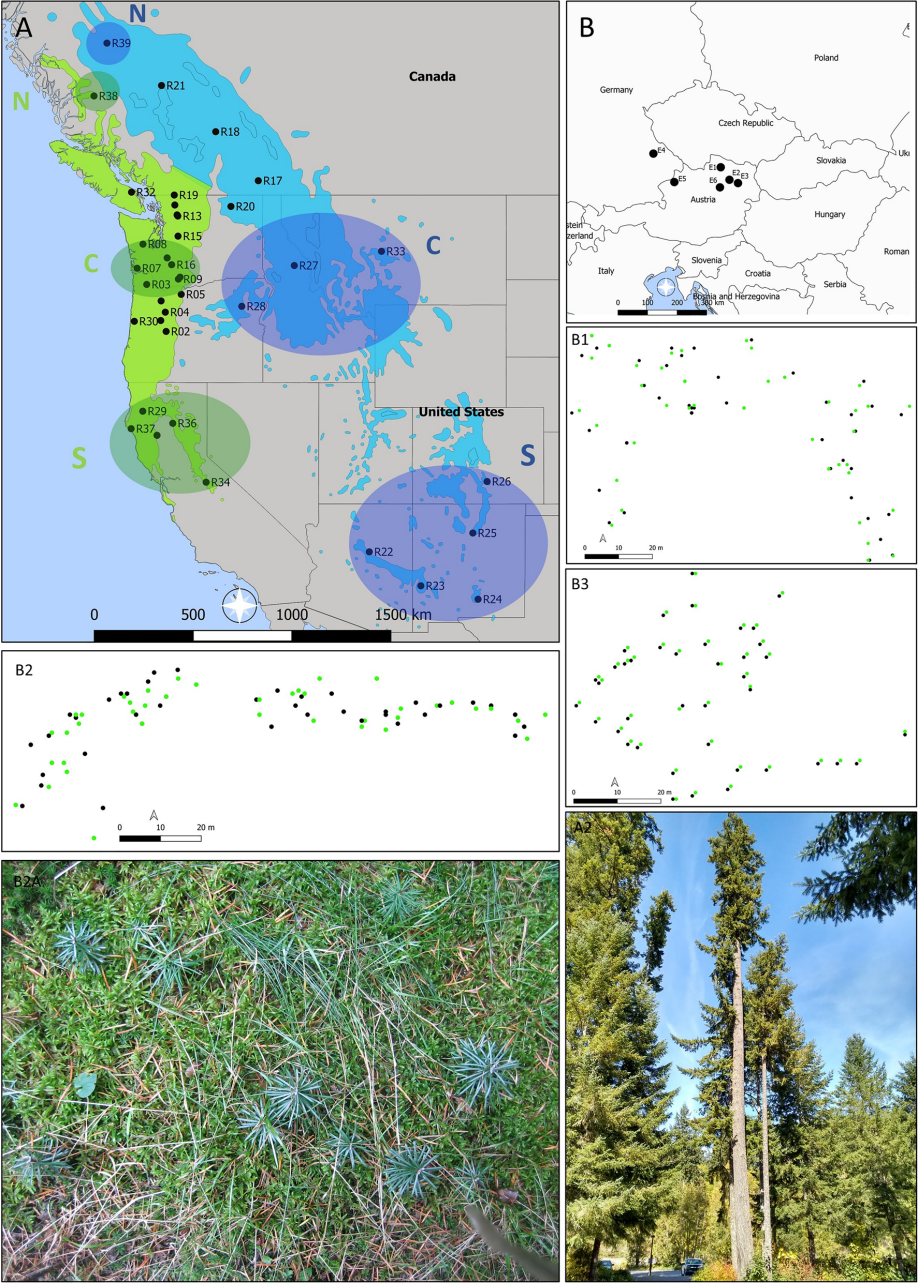
Native range of Douglas-fir with three reference genetic clusters (N-northern cluster, C- central cluster, S-southern) of coastal variety (marked by green) and three reference genetic clusters (N-northern cluster, C- central cluster, S-southern cluster) of interior variety (marked by blue) including reference populations (R01–R39) (**a**), distribution of studied populations in introduced range (**b**), distribution of old trees (black circles) and natural regeneration (green circles) in European populations E1–E3 (B1–B3), photographs of old growth in North America and natural regeneration in E3

In this study, we analysed climatic and genetic patterns of six old populations of Douglas-fir within their introduced range in Europe and compared them to 38 native populations in North America to shed light on dynamics of Douglas-fir in introduced range (Fig. 1). European adult populations were morphologically considered to be mixtures of coastal and interior varieties and contained natural regeneration. However, the exact geographic origin of the seed material used for their establishment and thus the variety composition was unknown. In these populations, we looked for signatures of possible hybridization and inter-varietal gene flow, differences in genetic diversity, fine-scale spatial genetic- and dispersal patterns, and climate similarities between non-native and native range.

We were specifically interested in answering the following questions:

1. Are both varieties present in old planted Douglas-fir populations and their natural regeneration? If so, from which geographic regions do they originate?
2. Do varietal hybrids exist within natural regeneration, and to which parental genotype can these be assigned to?
3. Do introduced and regenerated populations display founder effects (loss of genetic diversity) when compared to probable source populations and source varieties?
4. Do climatic conditions differ in the estimated native distribution areas of origin and the estimated area of introduction in Europe?

Simultaneously, we also tested the hypothesis that old European populations and European natural regeneration more likely originate from climatically similar source populations in North America. Furthermore, we investigated spatial genetic patterns in three populations in order to estimate propagule (pollen/seed) dispersal distances and differences in fine-scale spatial genetic structure (SGS) between old populations and natural regeneration expecting a lack of SGS in planted old stands, but presence of SGS in natural regeneration.

## Materials and methods

### Study species

Following its introduction to Europe in 1827 and planting in arboreta, botanical gardens and parks, Douglas-fir was introduced in forests at the end of the 19th, beginning of the 20th century and now grows on more than 823,534 ha occupying around 0.40% of European forest area (van Loo and Dobrowolska 2019). In its native range, in the western North America, Douglas-fir is divided into two geographically distinct varieties: *Pseudotsuga menziesii* var*. menziesii* or *viridis* (coastal variety), and *P. menziesii* var*. glauca* (interior variety, or Rocky-Mountain variety) (Fig. 1a). The latter, interior variety, occupies both a larger latitudinal range (≈ 4.500 km), and ecologically more diverse habitats (Lavender and Hermann 2014). In the native range, both varieties hybridize in contact zones situated in central Oregon (OR, U.S.) and in the northern Washington Cascades (WA, US) resulting in variety hybrids (van Loo et al. 2015; Hintsteiner et al. 2018). In British Columbia (BC, Canada), another contact zone developed into an extensive hybrid zone with an asymmetric introgression towards the coastal variety resulting in a wide range of inter-varietal admixed genotypes (Gugger et al. 2010; van Loo et al. 2015). The coastal variety from east of the Cascades in WA holds cold adapted alleles, which originate from this introgression with the interior Mountain variety (Eckert et al. 2009). The weak inter-varietal barriers were tested also artificially indenting to benefit from heterosis by combining adaptation to cold environment (typical for interior variety) with superior growth performance of the coastal variety (Rehfeldt 1979; Braun 1988, 1992). The superior growth and health performance of the coastal variety made this variety the preferred material for planting and cultivation in the majority of European countries. Only in northern Europe (Norway, Finland and Sweden), where extreme climate events are more dominant and compromise the faster growth potential of the coastal variety, the interior variety is superior to the coastal Douglas-fir (Magnesen 1987; Martinsson and Kollenmark 2001; Malmqvist et al. 2018).

### Study sites and sampling

We selected six planted forest populations of Douglas-fir in Europe which fulfilled the following three criteria: first, both Douglas-fir varieties were considered to be present based on the adult phenotypic variation; second, each population comprised more than 100 mature individuals, and third, natural regeneration of Douglas-fir existed. The geographic origin of the planted individuals was unknown. Five populations were located in Austria and one stand in Germany (Fig. 1b). The populations differed in age (between 30 and 85 years) and the composition of natural regeneration (Table 1). Three populations were younger than 50 years with natural regeneration represented by seedlings only, whereas three populations were older than 50 years with natural regeneration of different age classes. Within the populations, we randomly sampled plant material from up to 40 different adult individuals and their natural regeneration. Whenever possible, older individuals of natural regeneration were favored over younger offspring. In total, we obtained cambium samples from 236 adult trees and needles from 199 individuals of natural regeneration, respectively (Table 1). Collected plant material was directly placed into bags with silica gel. In three populations, coordinates of sampled old individuals and regeneration were recorded using a GPS devise (Fig. 1B1–B3).

**Table 1:** Geographical location of Douglas-fir populations and number (nu) of collected individuals (old trees and natural regeneration)

Stand	Country	Coordinates		Altitude	Adult trees		Young individuals	
					Nu	Age	Nu	Age class/age
E1	Austria	48.51°N	15.72°E	330–390	40	85	40	Different age classes/< 30
E2	Austria	48.52°N	15.76°E	440	39	> 50	32	Different age classes/< 20
E3	Austria	48.51°N	15.72°E	320–370	40	35	39	Seedlings/1–2
E4	GE	49.04°N	12.56°E	690	39	60	39	Different age classes/< 8
E5	Austria	48.24°N	13.45°E	415	39	30	12	Seedlings/1–2
E6	Austria	48.35°N	15.60°E	420	39	49	37	Seedlings/1–2
**Σ**	–	–	–	–	236	–	199	–

Geographical location includes decimal coordinates and altitude in m above sea level. Collected individuals are classified by age (in years) and age class

### DNA isolation and microsatellite genotyping

DNA was extracted from seedlings, needles or cambium tissue using OMEGA E.Z.N.A Plant DNA Kit (OMEGA Biotek, Inc. Norcross, Georgia, USA) according to manufacturer’s instructions. The extracted DNA was genotyped with 13 nuclear microsatellites (nu SSRs) following protocols and scoring analyses of van Loo et al. (2015). This marker set is identical to the one previously used for genotyping 766 individuals from 38 reference populations (RP) of Douglas-fir in the native range in Canada and the US (van Loo et al. 2015) as well as in introduced range when assessing the variety and geographic origin of introduced Douglas-fir (Eckhart et al. 2017; Hintsteiner et al. 2018).

### Variety composition (incl. presence of inter-varietal admixed individuals) and potential native origin

For estimation of variety composition and potential native origin we followed the hierarchical assignment analyses as described in van Loo et al. (2015) and Eckhart et al. (2017). In short, the variety composition was assessed using software STRUCTURE v.2.3.4. (Pritchard et al. 2000; Falush et al. 2003, 2007) and 38 reference populations from the native range of both varieties in Northwestern America (van Loo et al. 2015) (Fig. 1, Table S1). In the STRUCTURE analysis, 20 replicates of a run with a burn-in period of 500,000 and Markov chain Monte Carlo (MCMC) iterations of 1,000,000 were run for K = 2 under the admixture model with correlated allele frequencies (Falush et al. 2003). An individual was declared as coastal with *Qvar* (membership coefficient) > 0.80, interior with *Qvar* < 0.20 and inter-varietal admixed genotypes (hereafter also called variety hybrids) with 0.8 > *Qvar* > 0.2. (All threshold values originate from Eckhart et al. (2017) and are based on a simulation study used to assess the power of STRUCTURE admixture analysis to avoid false classification of parental and variety admixed genotypes. Eckhart et al. (2017) randomly selected subset of 120 coastal and 120 interior genotypes from the native range with the highest *Qvar* values. These were used to simulate genotypes of 120 F1 individuals with the HYBRIDLAB software v.1.1 (Nielsen et al. 2006). Following this procedure, which was replicated 5 times, all genotypes were used in STRUCTURE admixture analysis with K = 2 to estimate the cut-off values for parental and F1 hybrid genotypes).

Further, the potential geographic origin for the adult individuals and the natural regeneration of each stand were specified. When both varieties were present in the stand, these analyses were run separately for each variety. In general, analyses of geographic origin were run at two hierarchical levels. At the first level, the populations (and their varieties) were assigned to six robust reference genetic clusters (RGCs) defined by van Loo et al. (2015) (Fig. 1a), three of which represent the coastal variety (northern, central and southern coastal) and three the interior variety (northern, central, and southern interior). In STRUCTURE, these assignments were run under *locprior* option, with K = 3 for each variety. The remaining parameters were identical to those described above. At the second hierarchical level, the sampled populations were assigned to 38 reference populations (RPs) of the reference dataset (van Loo et al. 2015) using two-selected assignment measures in the software GeneClass2 (Piry et al. 2004) as applied by Eckhart et al. (2017) and Hintsteiner et al. (2018) to Douglas-fir of unknown origin. This included the use of two different statistical assignment measures; 1st Paetkau et al. (1995), and 2nd Rannala and Mountain (1997).

### Parentage analysis with focus on inter-varietal hybridization and propagule dispersal

Parent–offspring analysis was performed using program CERVUS 3.0.7 (Marshall et al. 1998). We used all adult parent genotypes as possible parents of the offspring. As several parent candidates can have a similarly high likelihood of parentage, CERVUS runs simulations of a random-mating population with the observed population’s allele frequencies to assign parentage at strict (95%) or relaxed (80%) confidence levels. In this study, we used only results of the strict assignments. The following parameters were set to run simulations and assignments: 10,000 simulated mating events, minimum of genotyped loci equals 9, genotyping error rate equals 0.01, and proportion of mistyped loci: 0.01. Results of parentage analyses and distances between geo-referenced trees of three populations (E1–E3) were further used to estimate minimum and maximum distances of realized propagule (pollen/seed) dispersal.

### Genetic diversity measures and founder effect

Standard descriptive genetic diversity measures were calculated for populations of European (all adult populations and their natural regeneration) and native range (allotted RPs, and RPs of allotted RGCs). Mean number of alleles (*N*_*a*_), observed heterozygosity (*H*_*o*_), expected heterozygosity (*H*_*e*_) and inbreeding coefficient (*Fis*) were analysed with the GenAlex v. 6.5 (Peakall and Smouse 2006, 2012). Allelic richness (*A*_*s*_) was calculated by rarefaction for a standardized populations size of 10 individuals using ADZE v. 1.0 (Szpiech et al. 2008). The Hardy–Weinberg (HW) exact tests were done to estimate the heterozygote deficiency with the software GENEPOP v. 4.1.4, using the default values (Raymond and Rousset 1995; Rousset 2008). To access a potential decrease of diversity, three different methods were used.

Firstly, two sample t-tests were used to test for differences of the genetic diversity between (1) European populations (six adult populations) versus American (38 populations), (2) European populations versus American populations to which European material was assigned to, (3) European populations versus American populations from the RGC with the largest diversity indices and (4) European populations only: six old populations versus six natural regenerations. Differences in both *A*_*s*_ and *H*_*e*_ were tested. In order to balance for differences in number of individuals and populations studied in Europe and America (comparisons 1–3), European populations were randomly divided into two subpopulations (with 20 individuals each) and diversity indices were estimated for each subpopulation before a mean value was calculated. Further, we randomly created 1000 new sub-groups from American data set (with six American populations for each sub-group) using a sub-sampling procedure. We then firstly performed a Shapiro–Wilk test to control for the normal distribution of these new variables before we run 1000 independent *t* tests between six European populations and the 1000 random sub-groups of American Douglas-fir populations (comparisons 1–3) using R.

Secondly, a one-tailed Wilcoxon test for heterozygote excess in European populations was run under the TMP mutation model in BOTLLENECK 1.2.02 (Cornuet and Luikart 1996) using run parameters of Mandák et al. (2013). And thirdly, the distribution of allele frequencies was tested for deviations from the L-shaped distribution as expected under the mutation-drift equilibrium using the mode-shift indicator in BOTLLENECK.

### Fine-scale spatial genetic structure

Spatial genetic structure (SGS) was assessed for three populations (E1–E3), for old and natural regeneration separately. We computed pairwise kinship coefficients (*F*_*ij*_) between individuals and their relationship with the spatial distance separating them using Spagedi 1.2 (Hardy and Vekemans 2002). Kinship coefficients were calculated for all pairs of individuals using the statistic of Loiselle et al. (1995) and 13 nuSSRs markers. In order to test for isolation by distance, we followed protocols of Vekemans and Hardy (2004), and van Loo et al. (2008), where pairwise kinship coefficients were regressed on the logarithm of spatial distance *d*_*ij*_, (*d* is the distance between *i* and *j*) to estimate the logarithmic regression slope *b*_*log*_. The significance of *b*_*log*_ was tested by permutating the spatial positions of individuals 10,000 times to obtain the frequency distribution of *b* under the null hypothesis that *F*_*ij*_ and *d*_*ij*_ were uncorrelated (cf. Mantel test). The extend of SGS was quantified using the *Sp* statistics of Vekemans and Hardy (2004) with *Sp* = −* b*_*log*_/(1 − *F*_(5,m)_). The *Sp* values allowed us a direct comparison of SGS between studied populations and with studies on other species. For graphical visualization of SGS in correlograms, average kinship coefficients were estimated for the following six distance classes (in meter): 0–5; 5–10; 10–20; 20–40; 40–80; 80–160.

### Climate comparison

**Fig. 2:**
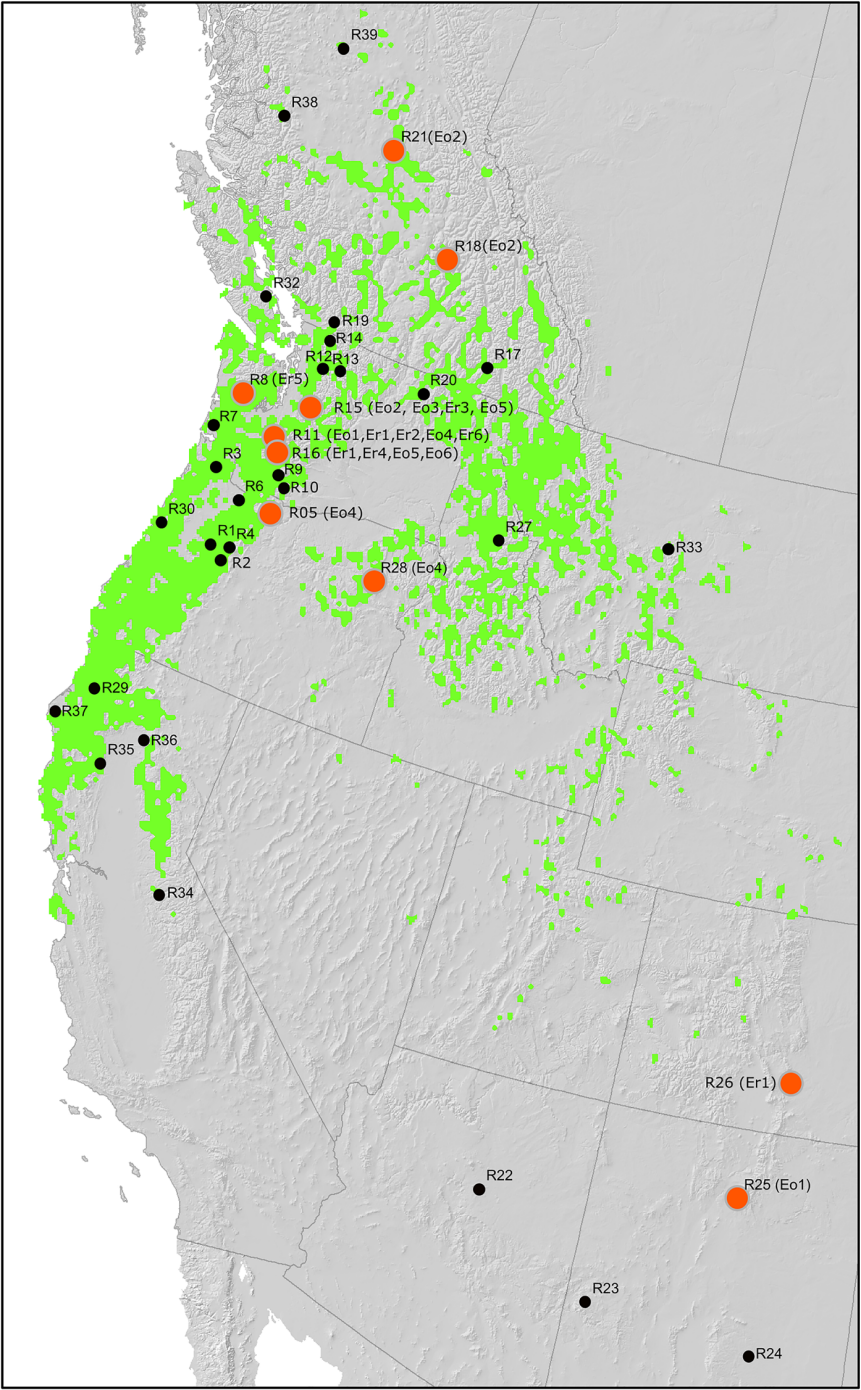
Genetic assignments of old growth (Eo1–Eo6) and natural regeneration (Er1–Er6) to reference populations (R01–R39) including native range drawn from the developed species distribution model (SDM)

Climate data for the climate comparison of the species seed origin in North America and the planted sites in Europe were obtained from the locally downscaled high-resolution climate models ClimateNA v5.21 (Wang et al. 2016) and ClimateEU v4.63 (Hamann et al. 2013) which contain in total 84 annual, seasonal and monthly climate parameters (Tave, Tmax, Tmin, Prec) as well as biologically relevant derived variables as for example positive and negative degree days, evaporation indices (Hamann and Wang 2012). In order to identify the most important climate variables, we developed a species distribution model (SDM) (Fig. 2) based on the correlation between the occurrence of Douglas-fir and the environment at its native range in North America using a Random Forest Classifier (Breiman 2001) as implemented in the Random Forest v.4.6-6 package (Liaw and Wiener 2002) of the R programming environment (R Core Team 2013). Current occurrence (presence and absence) of Douglas-fir was obtained from 71,182 inventory plots across North America from (Schroeder et al. 2010; Coops et al. 2011). Out of the 71,182 plots, Douglas-fir’s presence was observed in 12,469 plots and absence in 58,713 plots. Out of the climatic variables, the most influential variables explaining the current occurrence of Douglas-fir in North America were identified by the Recursive Feature Elimination (RFE) approach implemented with the “party” package (Strobl et al. 2009) of R. To further reduce the number of climate descriptors, we applied a principal component analysis. Pairwise climatic distances among North American as well as between North American and European populations were calculated on the basis of those principal components using Euclidian distances weighted for their Eigenvalue. Climate data analysis was performed with the statistic package Statistica 13.0 (TIBCO).

### Interaction of genetic and climatic distances

We tested if the realized pairwise climatic distances, i.e. distances between European populations and those North American populations that were assigned to be the most likely source populations, follow the expected climatic distance if seeds would have been drawn by chance from any of the putative seed origins. Both, expected and realized seed origins were drawn in histograms, and G-tests (McDonald 2014) were applied to check for significant differences in the frequency distribution of climatic distances.

## Results

### Varietal assignment, geographic origin and variety hybrids

The majority of individuals within the introduced range (407 individuals = 93.4%) and within individual populations (83.3–100%) were assigned to originate from the coastal variety (Table S2, Fig. S1). The presence of the interior variety was confirmed in three old populations (Eo1, Eo2, Eo4), and in the natural regeneration of one of them (Er1). Thus, only 1.8% of analysed individuals represent this variety. When compared to the interior variety, inter-varietal admixed individuals (variety hybrids) were present in a larger number of individuals (21 individuals; 4.8%) across five populations (five old populations and five natural regeneration).

Individuals assigned to the coastal variety were exclusively associated to the central reference genetic cluster (RGC) (*Q *= 0.75–0.95) which is situated in OR and WA (Table 2, Fig. 1a). On the contrary, individuals assigned to the interior variety, showed genetic signatures of all three RGCs in the native range and thus exhibit a broader latitudinal origin. More specifically, interior individuals of the populations Eo1 and its regeneration were found to originate from the southern RGC (*Q *= 0.94) located in the southern Rocky Mountains (in the US). Interior individuals of Eo4 originated from the central RGC (*Q *= 0.86), which is located in the Rocky Mountains of Idaho and Montana. And lastly, interior individuals from Eo2 were found to represent genotypes between the northern RGC and the central RGC with *Q* proportions of 0.56 and 0.36, respectively.

**Table 2:** Genetic assignments of old populations (Eo1–Eo6) and natural regeneration (Er1–Er6) after separating into varieties (C for coastal, I for interior)

Stand	Assignment to RGC (*Q*)	Assignment to RP (*Q*)	
		Paetkau et al.	Rannala & Mountain
Eo1/C	Central—C variety (95)	R11 (100)	R11 (100)
Eo1/I	Southern—I variety (90)	R25 (82.8)	R25 (99.92)
Er1/C	Central—C variety (90)	R16 (99.8)	R11 (100)
Er1/I	Southern—I variety (94)	R26 (99.1)	R26 (99.97)
Eo2/C	Central—C variety (88)	R15 (100)	R15 (99.4)
Eo2/I	Northern/central—I (56/36)	R21 (50.2)/R18 (49.5)	R18 (100)
Er2/C	Central—C variety (95)	R11 (100)	R11 (99.78)
Eo3/C	Central—C variety (94)	R15 (100)	R15 (99.98)
Er3/C	Central—C variety (79)	R15 (97.7)	R15 (100)
Eo4/C	Central—C variety (77)	R11 (99.8)	R11 (99.96)
Eo4/I	Central—I variety (86)	R05 (95.2)/R28 (2.0)	R28 (99.9)
Er4/C	Central—C variety (62)	R16 (97.4)	R16 (100)
Eo5/C	Central—C variety (86)	R15 (100)	R16 (100)
Er5/C	Central—C variety (75)	R08 (100)	R08 (100)
Eo6/C	Central—C variety (76)	R16 (100)	R16 (100)
Er6/C	Central—C variety (91)	R11 (100)	R11 (100)

Assignment results to reference genetic clusters (RGC) in STRUCTURE including mean cluster membership coefficient (*Q*) and to reference populations (RP): R01–R39 in GeneClass2 with likelihood scores in %

In assignments to reference populations (RPs), old populations with the coastal variety were assigned to R11, R15, or R16 located in the Western Cascades in Washington (Table 2, Fig. 2). The natural regeneration was assigned to the same RPs (R11, R15, or R16), but also to R08, which is located at the same latitude as R15, but somewhat closer to the coast. For the interior variety, trees from Eo1 were assigned to New Mexican populations (R25), whereas the natural regeneration of this stand had the highest likelihood to the neighbouring R26 from Colorado. Interior variety from Eo2 was assigned to Canadian Rocky Mountains (either R21 and R18 or R18 only depending on the method used) and interior variety from Eo4 had highest assignment scores to a coastal RP (R05) and to the neighbouring interior R28 located in the Blue Mountains of Oregon. Both assignment methods (“Paetkau”, and “Rannala & Mountain”) delivered matching assignment results for the natural regeneration and for four out of six old populations (Table 2).

### Parentage analysis with focus on inter-varietal hybridization and propagule dispersal

**Fig. 3:**
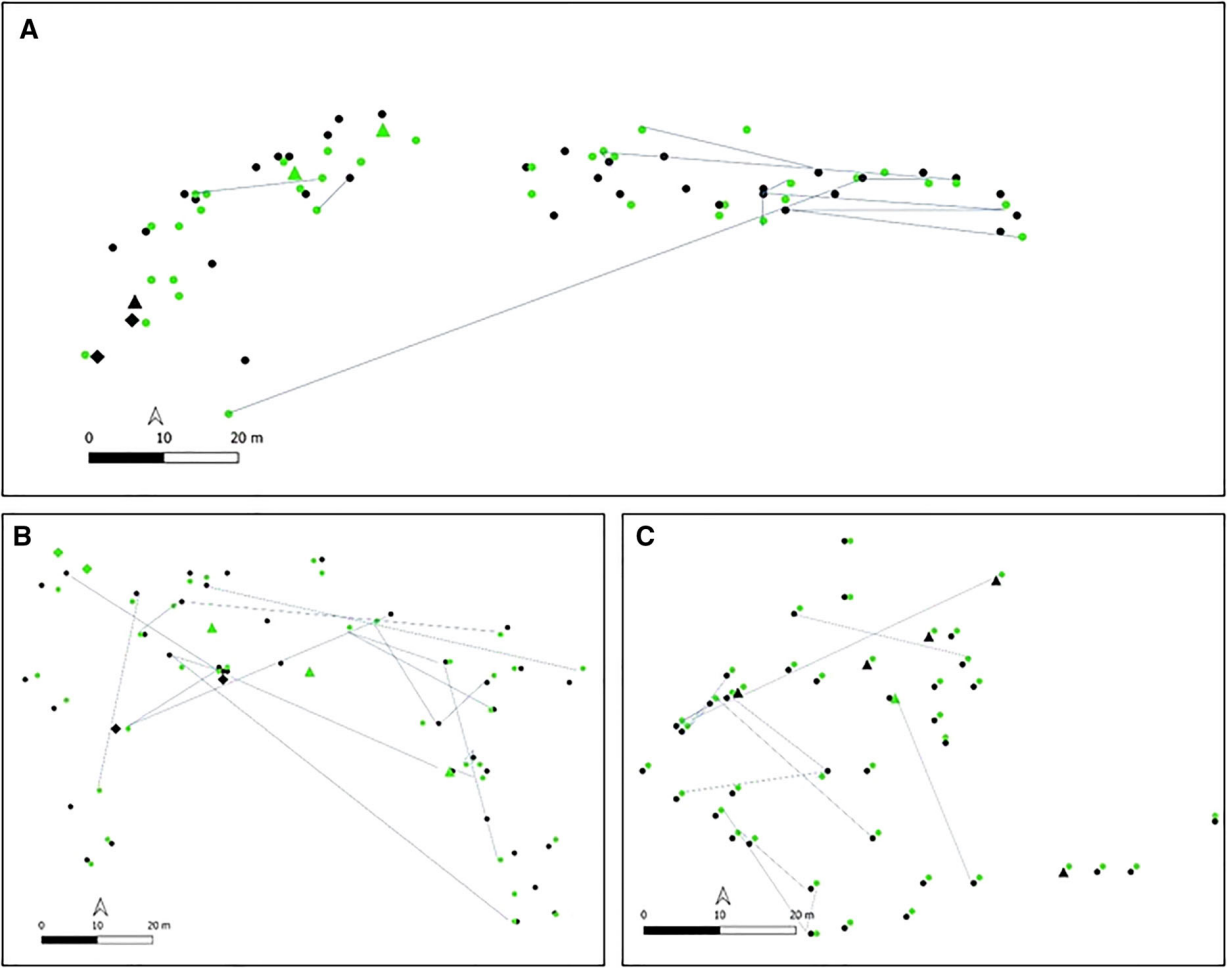
Parent-offspring relations (grey lines) between collected old trees (black color) and natural regeneration (green color) in introduced populations E1 (**b**), E2 (**a**), and E3 (**c**) represented by coastal variety (circle), interior variety (diamond), or admixed individuals (triangle), and with min. and max. distances (dispersal distances) between them in m: E1 (5–92), E2 (2.5–79), E3 (5–48)

Parent–offspring relations were found within each of the studied populations. In populations (1–4 and 6), 26–50% of the genotyped old trees contributed to the genotypes of 24–40% of the analysed offspring individuals (Table S3). In the youngest stand (E5), in which only 12 naturally regenerated individuals could be found, 11 of them (92%) carried genetic signatures of five parental genotypes (12.8%). Depending on the population, signatures of one parental genotype were found within one up to a maximum of four offspring (Fig. S2). Across all studied populations, the majority (49%) of parental genotypes contributed to one offspring, whereas fewer trees (13–23%) contributed to two, three and four offspring, respectively (Fig. S3). Although 11 inter-varietal admixed genotypes hybrids were found within natural regeneration, only to five of them a parental genotype could be assigned, which was in all five cases the coastal variety. Genotypic contribution of sampled interior variety was not identified. Propagule (pollen/seed) distances in populations E1–E3 ranged between 2.5 and 92 m (Fig. 3).

### Genetic diversity measures and founder effect

Tests of deviation from the Hardy–Weinberg equilibrium were all significant resulting in significantly positive *Fis* (Table S4). This result is identical to all previously published data on Douglas-fir. Except for Er6, where only 12 individuals could be analysed, the ranges of the diversity parameters (*H*_*e*_ and *As*_10_) for European populations were smaller (*H*_*e*_: 0.879–0.905, *As*_10_: 6.82–7.51) with slightly higher maximum diversity when compared to the corresponding American populations (*H*_*e*_: 0.629–0.904, *As*_10_: 6.67–7.48). Although European old growth and natural regeneration inhabited more diversity (*H*_*e*_ and *As*_10_) than the populations of associated interior RGCs, they were more similar to the populations of central RGS (with coastal variety) in both diversity indices (Table S4). The majority (88.6–100%) of population comparisons of *H*_*e*_ and *As*_*10*_ using subsampling procedure and 1000 independent t-tests were not significant. The largest number of significant differences (4.3% for *As*_10_, 11.4% for *H*_*e*_) we found in comparisons with subsampled groups of all North American populations, among which also interior populations with low diversity indices were present. Also the comparison of old populations with their natural regeneration revealed identical results, with no significant differences for *H*_*e*_ (t = 1.075, df = 10, *p* value = 0.308) and *As*_10_ (t = 1.4396, df = 10, *p* value = 0.1805). In addition, we found no evidence that the populations in Europe were exposed to strong bottlenecks in their recent history as no sign for heterozygote excess in populations and L-shaped distribution of allele frequencies were observed (data no shown).

### Fine-scale spatial genetic structure

Old populations and natural regeneration differed in SGS (Fig. S4, Table 3). As expected, analyses revealed consistently higher estimates of regression slopes *b*_*log*_ in natural regeneration (ranging from − 0.0062 to − 0.0136) than in old trees (*b*_*log*_ ranging from − 0.0018 to − 0.0081). These, however, were only significant in natural regeneration of E1 and E3. The strongest SGS was reported for Er1 as indicated by the over eight-times higher value of *Sp*. The correlograms reflected similar results. Natural regeneration of E1 (represented by young individuals of different age), and natural regeneration of E3 (represented exclusively by seedlings) showed spatial structuring (positive autocorrelations) at 3 m and 30 m, respectively. Random distribution of genotypes in planted old trees and natural regeneration of Er2 was reflected in non-significant kinship relationships along analysed distance classes.

**Table 3:** Summary of kinship autocorrelation (SGS) in old populations (Eo1–Eo3) and natural regeneration (Er1–Er3), including mean *F*_*ij*_ kinship values using the statistics of Loiselle et al. (1995) for the shortest distance interval (F_(5,m)_), the slope of the regression of mean kinship with the logarithm of spatial distance (b_log_) with standard errors and, the *Sp* statistic

Population	*F*_(5,m)_	*b*_*log*_ (± SE)	*Sp*
Eo1	0.0143	− 0.0018 n.s. (0.0020)	0.0018
Er1	0.1344	− 0.0136* (0.0030)	0.0158
Eo2	0.0171	− 0.0031 n.s. (0.0023)	0.0032
Er2	0.0226	− 0.0062 n.s. (0.0030)	0.0064
Eo3	0.0322	− 0.0081 n.s. (0.0034)	0.0083
Er3	0.0208	− 0.0092* (0.0024)	0.0094

n.s.—not significant, *P < 0.05

### Climate comparison and interaction of genetic and climatic distances

**Fig. 4:**
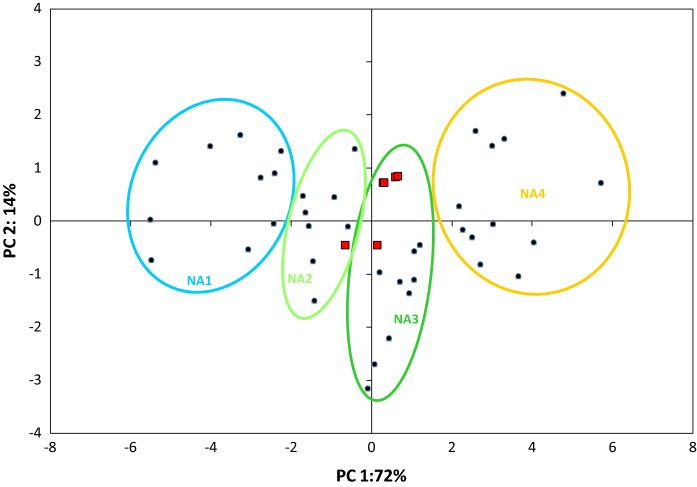
Clusters with groups of North American (NA1–NA4) populations (black circles) and European (EU) populations (red squares) as defined by PC1 and PC2 of the PCA

**Fig. 5:**
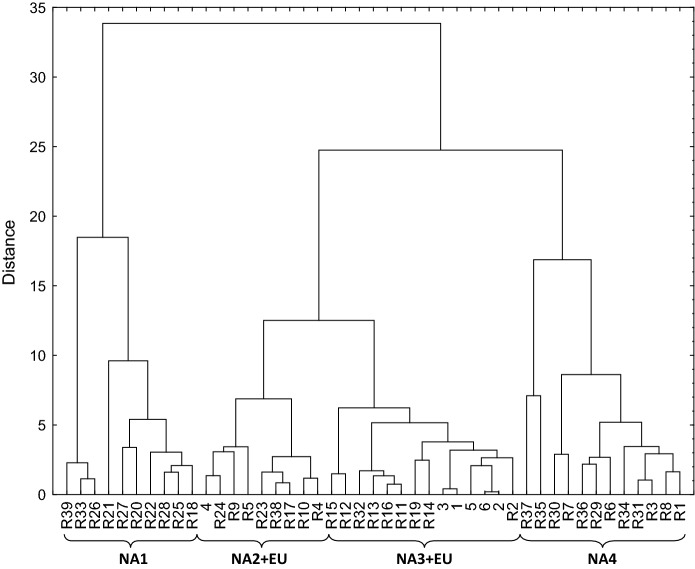
UPGMA-analysis of climate distances between 39 North American populations and six European populations on basis of the first four PCs of PCA explaining in total 97% of the variance of climate variability across reference populations

In order to identify the most important climate variables explaining the current occurrence of Douglas-fir in North America, the number of climate variables for a species distribution model was reduced with a RFE model to ten significant variables including the Julian date on which the frost-free period (FFP) begins (bFFP), the temperature difference between mean temperature of warmest and coldest month referred to as continentality (TD), the mean summer (May to Sept) precipitation (MSP), the precipitation as snow in mm between August in previous year and July in current year (PAS), the frost-free period (FFP), the Julian date on which FFP ends (eFFP), the degree-days below 0 °C (DD < 0), the extreme minimum temperature over 30 years (EMT), the mean temperature of the coldest month (MCMT), and the number of frost-free days (NFFD). The relative importance of each climate variable can be found in Table S5 in supporting Information. Using principal component analysis these ten were further reduced to four principal components (PC) explaining more than 97% of the climatic variability within the native range of Douglas-fir (Table S6). These four PCs allowed grouping of the potential seed origins into four climatically distinct groups in North America (Fig. 4): NA1 interior populations originating from southern to northern interior range—this climate in this wide regions is characterized by cold winters and high continentality; NA2 a geographically heterogeneous group containing provenances from the eastern Cascade range, the northern coastal range and the two most southern provenances; NA3 a geographically homogeneous group with populations from the western Cascade range; NA4 populations from the coastal and Californian range. Based on the climate comparison (Fig. 5), the analyzed European populations clustered mainly together with populations from NA3 (5 populations) and NA2 (one population).

**Fig. 6:**
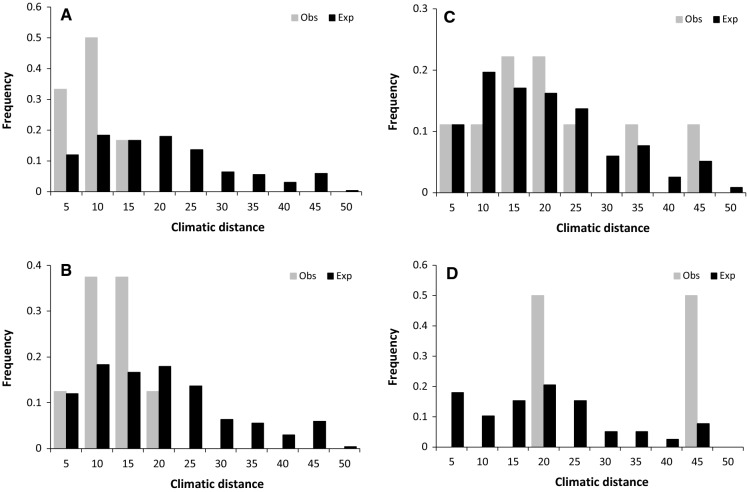
Frequency distribution of pairwise climate distances between all putative native populations in North America and the European stands (= Exp, expected distribution of climatic distances) and distribution of realized climatic distances, i.e. European stands and thus North American population that were assigned to be the most likely the seed origin of these stands (= Obs, observed distribution of climatic distances). The differences between expected and observed distances were compared by G-test. None of these test provided significant differences. Fig. a and c show relationships for old European trees assigned to coastal (**a**) and interior (**c**) North American populations, while b and d show the relationships for the young European regeneration assigned to coastal (**b**) and interior (**d**) populations

When we tested the realized climatic distances of European populations to their assigned source populations in North America, none of the observed climate distance distributions differed from the distance distribution expected by chance (Table S7, Fig. 6). Also, the mean climatic distances of the assigned genetic origin to adult European populations did differ only slightly from the assigned genetic origin of European natural regeneration (Table S8).

## Discussion

A smaller part of introduced tree species have been planted in forests, which among others ensure biodiversity, provide renewable resources for the bio-economy, and other ecosystem services (Castro-Diéz et al. 2019). While native tree species may serve these purposes without any problem, long generation cycles of the majority of introduced tree species are at the present associated with less knowledge, experience, higher risk and uncertainty about how the presence and invasion of these tree species may harm the native ecosystems in the future. Consequently numerous tree species have become controversial—especially when linking their potential or existing invasiveness and bio-economy. In our study we compared climatic and genetic patterns of populations from the native range and introduced range of Douglas-fir, known for both the increase of areas covered intentionally by this species in Europe and its adverse effects on biodiversity and habitats (Spiecker et al. 2019).

### Native range

The two varieties of Douglas-fir occupied different refugia during the Last Glacial Maximum (LGM) in America (Gugger and Sugita 2010; van Loo et al. 2015). Refugia in the Centre and South (Gugger and Sugita 2010; Gugger et al. 2010; van Loo et al. 2015) and possibly in the North (van Loo et al. 2015) of the current distribution range of each variety left a characteristic genetic imprint in the present genetic structure. Even after complex demographic processes and postglacial migration out of refugia (Gugger et al. 2010; Wei et al. 2011), three main genetic clusters (located in the North, the Centre and the South) are present in the native range of each variety. However, this phylogeographic pattern (geographical location of genetic clusters) is not identical with the climatic pattern in the different native parts. The 84 climatic parameters we used in this study divided the native range into 4 climatically groups (Figs. 4, 5). Most interestingly, NA1 has the widest geographic spread from New Mexico to northern BC, where the conditions of winter cold and temperature continentality were very similar. Group NA3 contained populations from the western Cascade range, whereas NA4 contained populations from mild conditions in California and in the coastal range of Oregon and Washington.

### Introduced range: variety, and geographic origin

The climate of the studied European introduced range was most similar to the climate of the group NA3, the western Cascade range (Figs. 4, 5), where the majority of the North American populations is situated to which coastal European Douglas-fir was assigned to (Table 2), but not the European interior variety.

The coastal variety, which prevails in the studied European populations, originated from a restricted area in Washington, US. This area is roughly 1° of latitude long and 2° of longitude wide, situated between 47.50° and 46.50°N (R15–R16), and 123.42°–121.55°E (R08–R15) in Washington US. Native populations from this area belong to the central coastal RGC, which derived from a well-characterised former refugium of the coastal variety on the Pacific coast in western WA and western OR (Wei et al. 2011). In addition, this RGC possesses the largest genetic diversity (expected heterozygosity, allelic richness) among all coastal RGCs and interior RGCs (van Loo et al. 2015). Also, a majority of other central European populations with coastal assignment (62 out of 67) originate from this particular central RGC (Hintsteiner et al. 2018). One possible explanation why the coastal variety from this particular area can be found most frequently in present old stands is associated to the settlement and logging/deforestation history in western North America which might have been the basis for continuous seed supply for the first half of the 20th century. However, also early knowledge about the outstanding growth performance of trees from this region (Schwappach 1911) or well-established seed harvest and trading routes might have limited the realized seed origin. This postulation is similar to that for *Prunus serotina* (black cherry). In introduced populations of this invasive tree in Europe, genetic imprints of native populations from the Allegheny plateau (an area in the east of the Appalachian mountain in the US) were identified (Pairon et al. 2010), where timber of this species was predominantly harvested (Marquis 1990).

The interior Douglas-fir was underrepresented within the studied populations, which matches both its lower ecological stability and low economic importance associated to the higher susceptibility to the fungus *Rhabdocline* needle cast (Stephan 1980) and poorer growth rates (Lavender and Hermann 2014). Nevertheless, the much smaller number of the interior variety trees originates from a much larger geographic area than the introduced coastal variety. The trees of interior variety contained genetic signature of all three genetic clusters and were assigned to the northern (R21, R18 in BC in Canada), central (coastal R5, interior R28) as well as to southern native reference populations in New Mexico (NM), U.S. (R25, R26) (Fig. 2).

### Introduced range: natural regeneration, dispersal patterns, SGS

Within the introduced range in Europe, the absence of a significant difference of expected heterozygosity and allelic richness between old growth and natural regeneration may be also explained by extensive seed- and pollen- flow. Revealed parent–offspring relations, where 26–50% (10–20) of old trees left their genetic imprint in 24–40% (9–16) of offspring confirmed the high contribution from outside of the genotyped parents. This may either predominantly originate from other parent pairs located within the stand as found in 71% of offspring studied by Fussi et al. (2013), or originate from other surrounding populations with a foreign pollen contribution of 70% (Valadon et al. 2010). Since we only analysed parent–offspring relationships and used bi-parentally inherited markers, it is not possible to dissect the realized propagule dispersal of 2.5–92 m (Fig. 3) into seed- and pollen- dispersal. For the biological invasion, the seed dispersal is of particular interest. In the wind pollinated Douglas-fir, around 80% of Douglas-fir seeds spread within a distance of 100–150 m from the maternal tree (Allen 1942; Dobbs et al. 1974; Barnhart et al. 1996). Thus, it is not surprising that in less than 30 years after plantation, *P. menziesii* invaded adjacent areas 100 m far away from the plantation in NE Spain (Brocano et al. 2005). The maximum seed dispersal distance, however, may reach 200 m (Eggert 2014), 800 m (Fowells 1965), or even 1–2 km (Dick 1955).

Studies analysing temporal changes of SGS indicate both trends: diluting over time (Chung et al. 2003, 2007; Zhou and Chen 2010) but also the opposite trend when SGS increases over time (Pardini and Hamrick 2008; Mandák et al. 2013; Berens et al. 2014). In our study, *Sp* values of all populations increased from old to natural regeneration resulting from spatial accumulation of related individuals in natural regeneration in close proximity of the mother trees. As expected for wind-pollinated trees of which seeds are predominantly dispersed by wind and gravity, which in addition have been most probably planted randomly during the population installment, the *Sp* estimates (0.0018–0.0083) of old populations were low, and they lacked any SGS. For comparison, extremely weak SGS (*Sp* estimates ranged from 0.00196 to 0.01076) was also found in *Larix laricina, Fraxinus excelsior*, *Pinus strobus*; all of them are wind-pollinated and wind-seed dispersed trees (Vekemans and Hardy 2004). SGS of natural regeneration varied from not present in regeneration of Er2 (*Sp* = 0.0064) to significant in Er3 (*Sp* = 0.0094) and Er1 (*Sp* = 0.0158), respectively. Although the SGS in Er3 may be explained by the spatial clustering of related seedlings, an explanation for the eight-times higher SGS in natural regeneration of S1 when compared to old trees of this population may be more complex. We analysed young trees of different age and thus most probably pooled life stages of different overlapping generations, which although common in tree species, here they may complicate and mislead interpretation of the SGS (Berens et al. 2014).

### Introduced range: variety hybrids

In natural regeneration, we identified 11 inter-varietal admixed genotypes. In five of them, only the coastal, but not interior Douglas-fir parent could be identified. Without detection of existing and contributing interior genotypes, the proof of inter-varietal hybridization within the studied populations is not reliable. The admixed genotypes could also originate from native areas, where both varieties come into contact (e.g. R15, R16, R5 in van Loo et al. 2015; Hintsteiner et al. 2018) and thus were already admixed before planted in Europe.

Also, none of the inter-varietal admixed individuals detected among the old trees contributed to the natural regeneration. This suggests that outcrossing in European Douglas-fir populations is high (as already discussed before) and that for more accurate parentage analysis more and at best all potential parents need to be genotyped to detect ongoing hybridization.

### Genetic variation

Although fast adaptation of introduced species is generally not limited to genetic variation (Bock et al. 2015; Dlugosch et al. 2015), the balance of evidence postulates that population bottlenecks (and genetic drift) negatively affects the spread of introduced species (Bock et al. 2015). In this study, the introduced old growth and natural regeneration were neither exposed to strong recent bottlenecks nor have been significantly different in genetic diversity to populations in the native range as revealed by the comparison to the entire native range and to the native populations from where they possibly originate. In studies on other introduced conifers such as *Pinus strobus* (Mandák et al. 2013), or *Cedrus atlantica* (Lefèvre et al. 2005) identical results to our study were presented for both genetic diversity comparisons and the absence of strong bottlenecks in introduced populations.

## Conclusions

Although both varieties of Douglas-fir are present in Europe, they revealed an opposite pattern in the prevalence of occurrence and the extent of the area where both varieties originate from. The coastal variety predominates in European forests, but individuals from the interior variety originate from a much larger geographic origin, spanning from the southern US to the interior BC (Canada). Interestingly, the extent of the range the interior Douglas-fir planted in Europe occupies in America is similar to the entire range which the coastal variety occupies in the native range (≈ 2.000 km). Variety hybrids and the coastal parents have been identified in the European populations, but additional sampling is required to identify the contributing interior variety to confirm inter-varietal gene flow in Europe as described in the native range (van Loo et al. 2015; Hintsteiner et al. 2018). Similarly to Douglas-fir, also other tree species such as *Ailanthus altissima* (tree of heaven), *Quercus rubra* (red oak), *Pinus strobus* (eastern white pine), or *Pinus contorta* (lodgepole pine) which have been reported to be invasive, are represented by ecologically different forms/varieties/subspecies. For these (forms/varieties/subspecies), we further lack information if they are present and if they cross within introduced continents.

The climatic distance between European populations and the assigned North American source populations vary broadly and suggest that European populations did not undergo strong climatic selection, in addition to the unaffected genetic diversity by introduction. Also, climatic distances to European old growth differ only slightly from distances to European natural regeneration indicating that neither reproduction nor natural regeneration strongly reduced the potential climatic space of the adult populations. Overall, it shows that climatic similarity (identical climatic niche) is only of minor importance for successful growth and reproduction when a conifer reaches a new range, which is in conclusion with provenance experiments and modeling of the species niche (Chakraborty et al. 2015, 2018) and climatic niche shifts in some introduced long-lived and invading woody plants (Camenen et al. 2016; Atwater et al. 2018). The genetic and combined genetic-climatic analyses we presented here including the estimation of geographic origin of introduced and invasive tree species shed light not only on establishment patterns after introduction such as propagule dispersal, regeneration composition, hybridization events, fine-scale spatial genetic structure, but also allow for refinement of climatic niche modeling (when using lower than species level).

## Electronic supplementary material

Below is the link to the electronic supplementary material.

Supplementary material 1 (DOCX 5108 kb)

## Data Availability

Data available from the Dryad Digital Repository: 10.5061/dryad.229m0tp.
