# Supplementary material for: North American Douglas-fir (P. menziesii) in Europe: establishment and reproduction within new geographic space without consequences for its genetic diversity
Source: Biol Invasions. 2019 Jul 8;21(11):3249–67. doi: 10.1007/s10530-019-02045-2 (PMC6936651; doi:10.1007/s10530-019-02045-2)
Supplement: Supplementary file 1 — Supplementary material 1 (DOCX 5108 kb) [file 10530_2019_2045_MOESM1_ESM.docx]

Supplementary material


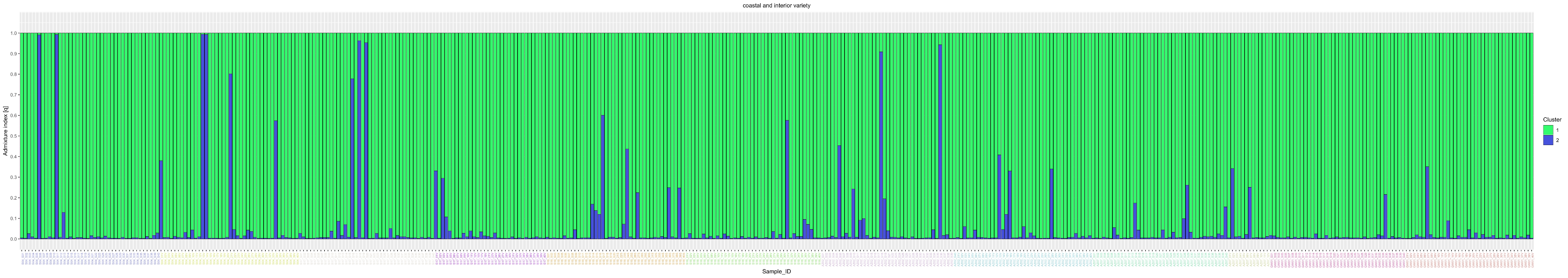


Eo1 Er1 Eo2 Er2 Eo3 Er3 Eo4 Er4 Eo5 Er5 Eo6 Er6

**Fig. S1** STRUCTURE results of variety and hybrid identification for K = 2 under the admixture model. Vertical lines delimit distinct genotypes of 431 individuals. Eo1- Eo6 and Er1-Er6 correspond to six populations of old trees (Eo) and natural regeneration (Er)

**Fig. S2** Number of parental genotypes per population (E1-E6) to which 1 - 4 offspring individuals (marked by diffent colors) were assigned to.

**Fig. S3** Number of parental genotypes (y axis) and % (within individual bars) to which 1 - 4 offspring individuals were assigned to.

**Fig. S4** Analysis of spatial genetic structure in old populations (Eo1-Eo3) and natural regeneration (Er1-Er3) by means of spatial autocorrelation analysis using kinship coefficients (*Fij*) of Loiselle et al. (1995). Correlograms show mean kinship coefficients (filled circles) between individuals for 6 different distance classes (m, x-axis). Broken lines delimit 95% confidence intervals defined through 10 000 permutations around the null hypotheses of random distribution of individuals in space. Kinship coefficients represented by red circles designate a greater genetic structure than expected at random (P < 0.005).


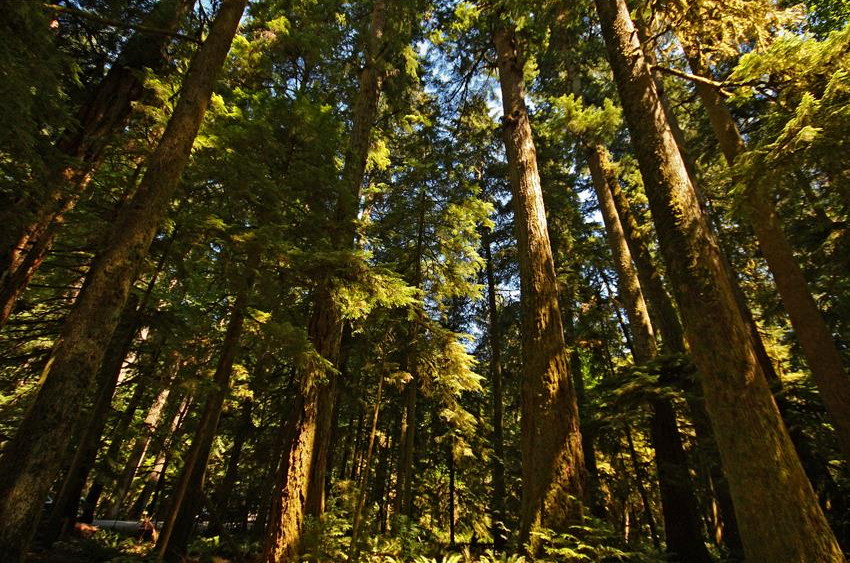


Fig. For social media (Douglas-fir old growth)

Supplementary material

**Table S1** Reference populations (Pop.-Nr.), geographic origin (state-prov., and coordinates inclusive altitude), number of collected individuals (N), membership to the genetic cluster I-IX (based on van Loo et al. 2015), label used for reference genetic cluster (RGC) in this study (N-northern cluster, C- central cluster, S-southern) and interior variety (N-northern cluster, C- central cluster, S-southern cluster)

| **Pop. -Nr.** | **State-Prov.** | **Lat.** | **Long.** | **Alt.[m]** | **N** | **Genetic Cluster** | **RGC** | |
| --- | --- | --- | --- | --- | --- | --- | --- | --- |
| R01 | US-OR | 44.41 | 122.47 | 525 | 20 | na |  | |
| R02 | US-OR | 44.22 | 122.07 | 975 | 20 | na |  | |
| R03 | US-OR | 45.77 | 123.22 | 200 | 20 | I | C-coastal v. | |
| R04 | US-OR | 44.50 | 122.00 | 975 | 20 | na |  | |
| R05 | US-OR | 45.40 | 121.38 | 975 | 20 | na |  | |
| R06 | US-OR | 45.38 | 122.30 | 250 | 20 | na |  | |
| R07 | US-WA | 46.37 | 123.73 | 45 | 18 | I | C-coastal v. | |
| R08 | US-WA | 47.25 | 123.42 | 100 | 20 | I | C-coastal v. | |
| R09 | US-WA | 45.97 | 121.53 | 675 | 20 | na |  | |
| R10 | US-WA | 46.04 | 121.44 | 1125 | 20 | I | C-coastal v. | |
| R11 | US-WA | 46.75 | 122.13 | 525 | 20 | I | C-coastal v. | |
| R12 | US-WA | 48.30 | 121.60 | 825 | 20 | I | C-coastal v. | |
| R13 | US-WA | 48.26 | 121.56 | 375 | 20 | I | C-coastal v. | |
| R14 | US-WA | 48.65 | 121.72 | 475 | 20 | na |  | |
| R15 | US-WA | 47.54 | 121.55 | 525 | 20 | I | C-coastal v. | |
| R16 | US-WA | 46.50 | 121.89 | 525 | 20 | I | C-coastal v. | |
| R17 | CA-BC | 49.50 | 117.27 | 825 | 20 | na |  | |
| R18 | CA-BC | 51.17 | 119.54 | 525 | 20 | na |  | |
| R19 | CA-BC | 49.00 | 121.75 | 475 | 20 | I | C-coastal v. | |
| R20 | US-WA | 48.60 | 118.73 | 750 | 20 | VI | C-interior v. | |
| R21 | CA-BC | 52.69 | 122.43 | 650 | 20 | na |  | |
| R22 | US-AZ | 34.93 | 111.35 | 2200 | 20 | VIII | S-interior v. | |
| R23 | US-NM | 33.43 | 108.60 | 2500 | 20 | VIII | S-interior v. | |
| R24 | US-NM | 32.83 | 105.55 | 2400 | 20 | VIII | S-interior v. | |
| R25 | US-NM | 35.75 | 105.83 | 2700 | 20 | VIII | S-interior v. | |
| R26 | US-CO | 37.95 | 105.07 | 3200 | 20 | VIII | S-interior v. | |
| R27 | US-ID | 46.47 | 115.35 | 900 | 20 | VI | C-interior v. | |
| R28 | US-OR | 44.95 | 118.15 | 1125 | 20 | VI | C-interior v. | |
| R29 | US-CA | 40.85 | 123.43 | 100 | 20 | na |  | |
| R30 | US-OR | 44.38 | 123.88 | 225 | 22 | na |  | |
| R32 | CA-BC | 49.10 | 124.03 | 450 | 20 | na |  | |
| R33 | US-MT | 46.99 | 110.70 | 1800 | 20 | VI | C-interior v. | |
| R34 | US-CA | 37.92 | 120.05 | 975 | 20 | III | S-coastal v. | |
| R35 | US-CA | 39.87 | 122.67 | 825 | 20 | IV | S-coastal v. | |
| R36 | US-CA | 40.36 | 121.83 | 975 | 22 | V | S-coastal v. | |
| R37 | US-CA | 40.14 | 124.05 | 825 | 22 | V | S-coastal v. | |
| R38 | CA-BC | 52.35 | 126.03 | 450 | 22 | II | N-coastal v. | |
| R39 | CA-BC | 54.04 | 125.34 | 850 | 20 | VII | C-interior v. | |
|  |  |  |  |  |  |  | |  |

na populations were assigned to represent cluster-mixed populations (for more details see van Loo et al. 2015)

**Table S2** *Q* values of STRUCTURE analysis for old populations (Eo1 – Eo6) and natural regeneration (Er1- Er6). *Q* values are displayed for individual level. Number of individuals and proportion (%) representing coastal variety (*Q >* 0.80), interior variety (*Q* < 0.20) and inter-varietal admixed individuals (0.80 > *Q* > 0.20)

| Population | *Q* at individual level | | | |  |
| --- | --- | --- | --- | --- | --- |
|  | *Q* < 0.20 | 0.80 > *Q* > 0.20 | *Q >* 0.80 | |  |
| Eo1 | 2 (5%) | 0 | | 38 (95%) |  |
| Er1 | 2 (5%) | 3 (7.5%) | | 35 (87.5%) | |
| Eo2 | 2 (5.1%) | 1 (2.6%) | | 36 (92.3%) | |
| Er2 | 0 | 2 (6.2%) | | 30 (93.8%) | |
| Eo3 | 0 | 5 (12.5%) | | 35 (87.5%) | |
| Er3 | 0 | 1 (2.6 %) | | 38 (97.4%) | |
| Eo4 | 2 (5.1%) | 2 (5.1 %) | | 35 (89.7 %) | |
| Er4 | 0 | 3 (7.7%) | | 36 (92.3 %) | |
| Eo5 | 0 | 1 (2.6 %) | | 38 (97.4%) | |
| Er5 | 0 | 2 (16.7 %) | | 10 (83.3 %) | |
| Eo6 | 0 | 1 (2.6%) | | 38 (97.4 %) | |
| Er6 | 0 | 0 | | 37 (100 %) | |
| **Σ** | 8 (1.8%) | 21 (4.8%) | | 407 (93.4%) | |

**Table S3** Number of assigned parent (old individuals) – offspring (natural regeneration) relations as revealed by parentage analysis within populations (E1-E6) using CERVUS

| populations | number of assigned individuals / in % | | | |
| --- | --- | --- | --- | --- |
|  | *parents* | | *offspring* | |
| E1 | 20 / 50% | 16 / 40% | |  |
| E2 | 15 / 38.5% | 13 / 33.3% | |  |
| E3 | 15 / 37.5% | 15 / 38.5% | |  |
| E4 | 11 / 28.2% | 10 / 25% | |  |
| E5 | 13 / 33.3% | 11 / 91.7% | |  |
| E6 | 10 / 25.6% | 9 / 24.3% | |  |

**Table S4** Genetic diversity parameters for six old European populations (Eo1-Eo6), their natural regeneration (Er1-Er6) and for reference genetic clusters (RGC) and reference populations (RP) from North America. American populations were represented by 20 individuals.

| Population | *Na* | | *H_O_* | *H_E_* | | *F_IS_* | *A_S10_* | |
| --- | --- | --- | --- | --- | --- | --- | --- | --- |
| Eo1 | 21.769 | | 0.662 | 0.906 / *0.888 | | 0.271 | 7.39 / *7.21 | |
| Er1 | 19.692 | | 0.711 | 0.913 | | 0.221 | 7.22 | |
| Eo2 | 22.231 | | 0.686 | 0.913 / *0.895 | | 0.248 | 7.40 / *7.33 | |
| Er2 | 19.077 | | 0.669 | 0.913 | | 0.267 | 7.40 | |
| Eo3 | 20.846 | | 0.728 | 0.902 / *0.879 | | 0.190 | 7.18 / *6.82 | |
| Er3 | 21.462 | | 0.741 | 0.903 | | 0.175 | 7.24 | |
| Eo4 | 21.923 | | 0.718 | 0.915 / *0.899 | | 0.214 | 7.51 / *7.41 | |
| Er4 | 19.154 | | 0.751 | 0.904 | | 0.167 | 7.18 | |
| Eo5 | 22.462 | | 0.679 | 0.920 / *0.905 | | 0.260 | 7.60 / *7.51 | |
| Er5  Eo6  Er6 | 7.462  23.385  20.923 | | 0.604  0.708  0.717 | 0.805  0.909 / *0.892  0.913 | | 0.255  0.220  0.213 | 5.47  7.56 / *7.44  7.42 | |
| RGC central interior v. | - | | - | 0.841-0.880 | | - | 6.65-7.15 | |
| RGC southern interior v. | - | | - | 0.629-0.808 | | - | 6.66-6.70 | |
| RGC central coastal v. | - | | - | 0.870-0.904 | | - | 6.94-7.65 | |
| R05 coastal variety | 15.692 | | 0.670 | 0.891 | | 0.245 | 7.48 | |
| R08 coastal variety | 14 | | 0.677 | 0.880 | | 0.226 | 7.06 | |
| R11 coastal variety | 15.769 | | 0.753 | 0.904 | | 0.168 | 7.65 | |
| R15 coastal variety | 15.846 | | 0.711 | 0.887 | | 0.198 | 7.43 | |
| R16 coastal variety | 15.154 | | 0.691 | 0.889 | | 0.221 | 7.16 | |
| R18 interior variety | 14.154 | | 0.543 | 0.884 | | 0.389 | 7.29 | |
| R21 interior variety | 12.692 | | 0.607 | 0.870 | | 0.304 | 6.88 | |
| R24 interior variety | 10.308 | | 0.491 | 0.629 | | 0.229 | 6.67 | |
| R25 interior variety | 15.308 | | 0.624 | 0.808 | | 0.247 | 7.03 | |
| R26 interior variety | 15.409 | | 0.517 | 0.754 | | 0.354 | 7.04 | |
| R28 interior variety | 12.769 | | 0.545 | 0.853 | | 0.372 | 7.08 | |
|  | |  |  | |  |  | |  |
|  | |  |  | |  |  | |  |

Included are: the mean of number of alleles (*Na*), observed heterozygosity (*Ho*), expected heterozygosity (*He*), inbreeding coefficient (*Fis*) and allelic richness (*As_8_*) standardized for a population size of 10 individuals

* mean value calculated for European populations after dividing them into two subsampling populations (20 and 20 individuals)

**Table S5** The relevance of the climate parameters used to develop the Random forest SDM with Douglas-fir occurrence in North America. The relative importance is permuted as a measure of the decrease in model accuracy. It is determined during the out of bag error calculation phase. The more the accuracy of the random forest decreases due to the exclusion (or permutation) of a single variable, the more important that variable is deemed, and therefore variables with a large mean decrease in accuracy are more important for classification of the data

| **Acronym** | **Climate variable** | **Mean Decrease Accuracy** |
| --- | --- | --- |
| MSP | mean summer (May to Sept) precipitation | 157.16 |
| TD | the temperature difference between the mean temperature of warmest and coldest month referred to as continentality | 137.61 |
| PAS | precipitation as snow in mm between August in the previous year and July incurrent year | 114.08 |
| bFFP | the Julian date on which the frost-free period (FFP) begins | 109.46 |
| EMT | extreme minimum temperature over 30 years | 95.65 |
| DD<0 | degree-days below 0°C or the chilling degree-days | 86.47 |
| MCMT | mean temperature of the coldest month | 69.04 |
| eFFP | the Julian date on which FFP ends | 63.26 |
| FFP | frost-free period | 61.54 |
| NFFD | the number of frost-free days | 54.36 |

**Table S6** Contribution of climate variables to the four principal components describing the climate in the natural range of Douglas-fir and the climatic distances between populations

| Variable |  | PC1 | PC2 | PC3 | PC4 |
| --- | --- | --- | --- | --- | --- |
| MCMT | Mean temperature of the coldest month | 0.946 | -0.084 | -0.224 | -0.144 |
| TD | Continentality | -0.769 | 0.503 | 0.220 | 0.211 |
| MSP | Mean summer (May to Sept) precipitation | -0.102 | -0.814 | 0.555 | -0.121 |
| DD<0 | Degree-days below 0°C | -0.909 | 0.154 | 0.145 | 0.171 |
| NFFD | Number of frost-free days | 0.986 | -0.004 | 0.025 | 0.114 |
| bFFP | Julian date on which FFP begins | -0.884 | -0.183 | -0.341 | -0.239 |
| eFFP | Julian date on which FFP ends | 0.980 | 0.044 | 0.036 | 0.126 |
| FFP | Frost-free period | 0.952 | 0.120 | 0.202 | 0.191 |
| PAS | Precipitation as snow between August of the previous and July of the current year | -0.518 | -0.623 | -0.313 | 0.491 |
| EMT | Extreme minimum temperature over 30 years | 0.972 | -0.119 | -0.150 | 0.068 |
|  |  |  |  |  |  |
| Eigenvalue |  | 7.154 | 1.399 | 0.707 | 0.478 |
| Variance explained (%) | | 71.538 | 13.989 | 7.068 | 4.781 |

**Table S7** G-test for comparing the frequency distribution of pairwise climate distances between all putative source populations in North America and the European populations with the distribution of observed climatic distances, i.e. European populations and thus North American population that were assigned to be the most likely seed origin of these populations

| Comparison | p-value |
| --- | --- |
| Interior NA vs. adult populations EU | 0.24 |
| Interior NA vs. natural regeneration EU | 0.39 |
| Coastal NA vs. adult populations EU | 0.17 |
| Coastal NA vs. natural regeneration EU | 0.48 |

**Table S8** Mean climatic distances between European populations and the assigned source populations in North America

|  | Adult European | Natural regeneration |
| --- | --- | --- |
| Interior | 12.04 | 9.71 |
| Coastal | 11.22 | 10.78 |
